# A comparison of organ preservation in older adults with stage I rectal cancer

**DOI:** 10.1007/s00384-025-04940-8

**Published:** 2025-07-01

**Authors:** Annmarie Butare, Scarlett Hao, Anas Taha, Michael Drew Honaker

**Affiliations:** 1 Department of Surgery, East Carolina University Brody School of Medicine, Greenville, NC USA; 2 Department of Biomedical Engineering, Faculty of Medicine, University of Basel, Basel, Switzerland; 3 Department of Surgery, Division of Surgical Oncology, East Carolina University Brody School of Medicine, Greenville, NC USA

**Keywords:** Rectal neoplasms, Organ preservation, Aged, Social determinants of health

## Abstract

**Background:**

Total mesorectal excision (TME) remains the primary recommended treatment for high-risk T1 and T2 rectal cancer. However, growing evidence suggests preoperative therapy may lead to eligibility for organ preservation (OP), avoiding the morbidity of major resection, which may be beneficial in older adults. The primary aim of the study was to compare rates of OP in adults 70 years of age and older to those less than 70 with T1 lesions rectal cancers with high-risk features and T2 rectal cancers.

**Methods:**

A retrospective, cohort study of patients with high-risk stage 1 rectal cancer was identified within the National Cancer Database (NCDB). Primary outcome was the association of age with receipt of organ preservation. Multivariate analysis was conducted to examine the effect of covariates on the outcome.

**Results:**

Out of 38,714 patients, 34.4% were ≥ 70 years, 42.3% were female, and 75.6% had a Charlson Deyo comorbidity score of 0. Older adults were more likely to received OP compared to younger patients (45.6% vs 30.6%, *p* < 0.001). This persisted on adjusted analysis (OR 1.9, *p* < 0.001). Other factors predictive of receiving OP include non-Hispanic Black race/ethnicity (OR 1.5, *p* < 0.001), lack of insurance (OR 1.5, *p* < 0.001), increased comorbidity score (OR 1.7 for CDCC of 3, *p* < 0.001), treatment at a community facility compared to academic facility (OR 1.4, *p* < 0.001), and female sex (OR 1.2, *p* < 0.001).

**Conclusions:**

Although current guideline recommendations for high-risk T1 and T2 rectal cancer is TME, a significantly higher proportion of older adult patients undergo organ preservation. This is more pronounced in comorbid and disadvantaged patients.

## Introduction

As the treatment of rectal cancer continues to evolve, there is growing interest in organ preservation (OP) approaches based on recent data from randomized trials showing increased rates of complete clinical response (cCR) with minimal to no detriment in oncologic outcomes [1, 2]. Compared to total mesorectal excision (TME), OP offers the benefit of morbidity reduction, including a reduced incidence of symptoms associated with low colorectal or coloanal anastomoses and/or a potential permanent stoma. Particular populations, such as older adults, are at increased risk of postoperative complications, and thus may have greater benefit from an OP approach [3, 4].

Current guidelines for patients with high risk T1 and T2 stage I rectal cancer is oncologic resection without neoadjuvant therapy [5], while the majority of studies examining organ preservation rates (OPR) only include patients with stage II/III rectal cancer [6, 7]. For patients with early cancer who may benefit from avoiding TME, such as those of advanced age or have significant comorbidities, an argument can be made for to undergo neoadjuvant therapy with the goal of achieving a cCR and avoidance of oncologic resection.

The primary aim of the current study was to examine the utilization of OP approaches in older adult patients with high-risk stage I disease compared to younger patients. We hypothesize that older adult patients are more likely to undergo an OP approach than younger patients secondary to increasing comorbidities associated with advanced age. Secondarily, we performed a subgroup analysis of older adults to evaluate factors associated with OP in this cohort.

## Methods

### Data source and study population

A retrospective, observational study was conducted utilizing the National Cancer Database (NCDB). The NCDB was established in 1989 as a joint project by the American College of Surgeons and the American Cancer Society [8]. It is a nationwide, comprehensive, clinical surveillance resource that currently captures 72% of all newly diagnosed malignancies in the USA. Patients with adenocarcinoma of the rectum diagnosed between 2004 and 2020 from the NCDB were identified. Patients over 18 years of age with clinical T2 lesions and clinical T1 lesions with one of the following high-risk pathologic features: perineural invasion (PNI), lymphovascular invasion (LVI), and grade 3 or 4 (poor or anaplastic) differentiation, were included in the study. Patients were excluded if they did not receive any type of treatment, had missing staging or treatment data, and histology of cancer other than adenocarcinoma. This study was considered nonhuman subject research by the Institutional Review Board (IRB) due to its use of public, deidentified data. Results are reported according to the Strengthening the Reporting of Observational Studies in Epidemiology (STROBE) reporting guidelines [9].

### Variables and outcomes

Variables analyzed from the Participant User File (PUF) included patient age, sex, race and ethnicity, facility type, Charlson-Deyo Comorbidity Index (CCI), residential setting (metro, urban, rural), payor type, percent without high school degree in the region of residence, clinical TNM classification as defined by the American Joint Commission on Cancer (AJCC) manual based upon year of diagnosis, receipt what of neoadjuvant chemotherapy or radiation therapy, receipt of surgery, and type of surgery performed. To define the age cohorts, older adult was defined as $\ge$ 70 years of age. As the proportion of older individuals increases in the USA, there is variability as to the definition of an older patient. Seventy years was selected based upon other studies of older adults, which define this to be between 65 and 75 years [10, 11]. The primary outcome of interest was treatment by OP versus TME by age cohort. OP was defined as receiving chemotherapy or radiation therapy followed by either no surgery or local excision. TME was defined by receipt of any oncologic resection with or without neoadjuvant or adjuvant therapy. Covariates that were predictive of OP overall were examined. In addition, a subgroup analysis was conducted to evaluate factors associated with OP specifically within the older adult cohort.

### Statistical analysis

Continuous variables are summarized by median and interquartile range (IQR). Categorical variables are summarized by the number of patients and percent for each category. Chi square tests were conducted to evaluate significance in categorical data, and the Wilcoxon rank sum test was used to compare medians across cohorts. Missing data was treated as missing, and no method of imputation was planned.

Univariate logistic regression was performed to assess for the outcome of OP compared to TME using variables chosen a priori based on clinical relevance, followed by a variable selection process using only predictor variables found to be statistically significant to build the final binary logistic regression model with the best predictive fit based on the Hosmer–Lemeshow test. The final model included age cohort, sex, race/ethnicity, payor, facility type, percent without high school degree, CCI, and rurality. Analyses were performed with the SPSS statistical software (version 29, IBM Corporation, Armonk, NY). Statistical tests were two-sided, with *p*-value < 0.05 considered a significant finding.

## Results

### Demographics

Of the initial 387,099 patients with rectal cancer, 38,714 met inclusion criteria. Overall, the study population had a median age of 64 years (IQR 54–74) with 34.4% ≥ 70 years of age. Demographic data for each age cohort are shown in Table 1. Older adult patients were more likely to be non-Hispanic White (85.28% vs 79.32%) and were more commonly treated at comprehensive cancer centers (42.42% versus 29.27%). Younger patients were treated at similar rates between comprehensive and academic centers (36.95% and 36.93%, respectively). Expectedly, a larger proportion of the older adult cohort were Medicare-insured (87.08% compared to 22.25% of the non-elderly cohort) and had a higher comorbidity index (67.11% with a score of 0 compared to 80.11% of the younger cohort).

**Table 1:** Cohort demographics

Variable	Category	Non-elderly (*n* = 24,268)	Elderly (*n* = 12,661)	*p*-value
Age (years)		57 (50–63)	77 (73–83)	
Sex	Male	14,768 (58.14%)	7573 (56.88%)	0.017
	Female	10,632 (41.86%)	5741 (43.12%)	
Race/ethnicity	Non-Hispanic White	19,249 (79.32%)	10,797 (85.28%)	< 0.001
	Non-Hispanic Black	2228 (9.18%)	856 (6.76%)	
	Hispanic	1572 (6.48%)	554 (4.38%)	
	AAPI	1077 (4.44%)	415 (3.28%)	
	Other	142 (0.59%)	39 (0.31%)	
Facility type	Academic	8901 (36.93%)	3897 (29.27%)	< 0.001
	Community	1626 (6.75%)	1112 (835%)	
	Comprehensive	8906 (36.95%)	5648 (42.42%)	
	Integrated	4667 (19.37%)	2657 (19.96%)	
Rurality	Metro	20,474 (83.82%)	10,582 (82.04%)	< 0.001
	Urban	3537 (14.48%)	2038 (15.80%)	
	Rural	414 (1.69%)	279 (2.16%)	
Payor	Private	16,035 (64.21%)	1330 (10.12%)	< 0.0001
	Medicare	5557 (22.25%)	11,447 (87.08%)	
	Medicaid	2024 (8.11%)	190 (1.45%)	
	Uninsured	956 (3.83%)	55 (0.42%)	
	Other governmental	400 (1.60%)	123 (0.94%)	
% No HSD	< 5%	4934 (22.04%)	2523 (21.12%)	0.014
	5–9%	6553 (29.27%)	3628 (30.36%)	
	9.1–15.2%	6223 (27.79%)	3406 (28.51%)	
	> 15.3%	4680 (20.90%)	2391 (20.01%)	
CDCC	0	20,349 (80.11%)	8935 (67.11%)	< 0.001
	1	3722 (14.65%)	2819 (21.17%)	
	2	825 (3.25%)	981 (7.37%)	
	3 +	504 (1.98%)	579 (4.35%)	

*Abbreviations*: *AAPI* Asian American and Pacific Islander, *HSD* high school degree, *CDCC* Charlson-Deyo Comorbidity Index

### Rates of organ preservation

Overall, the majority of the study population underwent TME (Table 2). A higher proportion of the younger age cohort underwent TME (69.5% vs 54.5%). Of the patients who received OP, 67.4% patients underwent neoadjuvant therapy alone and 32.6% underwent neoadjuvant therapy with local excision. This distribution was similar for both age cohorts.

**Table 2:** Cohort treatment approach

	Non-elderly	Elderly	Total	*p* < 0.001
TME	17,640 (69.45%)	7250 (54.45%)	24,890	
OPR	7760 (30.55%)	6064 (45.55%)	13,824	
TOTAL	25,400	13,314	38,714	

### Regression analysis

On univariate regression, all tested variables had statistical significance in at least one category. On multivariate regression, older adult patients were more likely to be treated with an OP approach (OR 1.76, 95% CI 1.65–1.87; *p* < 0.01) compared to younger patients. Female sex, non-Hispanic black race/ethnicity, non-private payor type, treatment at a community facility, a comorbidity score of 3 or greater, and residing in a region within the fourth quartile of residents without a high school degree were also associated with a higher likelihood of undergoing an OP approach compared to their respective reference categories (Table 3). Notably residing in an urban area and a lower comorbidity score had decreased odds of OP.

**Table 3:** Multivariable regression analysis for odds of organ preservation

Variable	Category	OR	*p*-value	95% CI
Gender	Female	1.178	< 0.001	1.122–1.236
Race/ethnicity (ref: Non-Hispanic White)	Black	1.486	< 0.001	0.362–1.621
	Hispanic	1.009	0.877	0.905–1.123
	AAPI	0.832	0.004	0.733–0.944
	Other	1.309	0.117	0.935–1.832
Facility type (ref: Academic)	Community	1.428	< 0.001	1.299–1.569
	Comprehensive	0.981	0.516	0.927–1.039
	Integrated	0.953	0.174	0.888–1.022
Rurality (ref: metro)	urban	0.83	< 0.001	0.773–0.891
	rural	0.95	0.576	0.795–1.136
Payor (ref: private)	Medicare	1.203	< 0.001	1.129–1.283
	Medicaid	1.321	< 0.001	1.187–1.471
	Uninsured	1.505	< 0.001	1.295–1.750
	Other Governmental	1.829	< 0.001	1.492–2.241
% No HSD (ref: < 5%)	5–9%	1.012	0.74	0.945–1.084
	9.1–15.2%	0.976	0.508	0.910–1.048
	> 15.3%	1.092	0.025	1.011–1.180
CDCC (ref: 0)	1	0.864	< 0.001	0.809–0.921
	2	0.925	0.177	0.827–1.036
	3 +	1.404	< 0.001	1.221–1.613
Age group	Older adult	1.758	< 0.001	1.651–1.871

Abbreviations: *AAPI* Asian American and Pacific Islander, *HSD* high school degree, *CDCC* Charlson-Deyo Comorbidity Index

### Subgroup analysis

On multivariate regression analysis in patients 70 years of age or greater, female sex remained a significant predictor of undergoing an OP approach (OR 1.22, 95% CI 1.13–1.32; *p* < 0.001). Other covariates associated with an increased odds of undergoing an OP approach included non-Hispanic black race/ethnicity, treatment at a community program, and having a higher CCI (Table 4). Urban residence was associated with a lower likelihood of being treated with an OP approach compared to those residing in a metro residence.

**Table 4:** Multivariable regression analysis for odds of organ preservation in older adults

Variable	Category	OR	*p*-value	95% CI
Sex	Female	1.223	< 0.001	1.133–1.320
Race/ethnicity (ref: Non-Hispanic White)	Black	1.201	0.020	1.029–1.401
	Hispanic	0.978	0.814	0.978–1.178
	AAPI	0.916	0.414	0.741–1.132
	Other	1.296	0.445	0.666–2.525
Facility type (ref: Academic)	Community	1.223	< 0.001	1.133–1.320
	Comprehensive	0.984	0.731	0.898–1.078
	Integrated	0.994	0.920	0.889–1.112
Rurality (ref: metro)	Urban	0.811	< 0.001	0.728–0.904
	Rural	1.004	0.978	0.772–1.306
% No HSD (ref: < 5%)	5–9%	0.988	0.832	0.888–1.101
	9.1–15.2%	0.970	0.585	0.869–1.083
	> 15.3%	1.082	0.205	0.958–1.223
CDCC (ref: 0)	1	0.962	0.421	0.875–1.057
	2	0.980	0.792	0.846–1.136
	3 +	1.520	< 0.001	1.257–1.838

*Abbreviations*: *AAPI* Asian American and Pacific Islander, *HSD* high school degree, *CDCC* Charlson-Deyo Comorbidity Index

## Discussion

Organ preservation in patients with rectal cancer arose from quality-of-life outcomes associated with low anterior resections, coloanal anastomoses, and permanent end colostomies. In this large database study of over 30,000 patients with high risk T1 and T2 rectal adenocarcinoma, older adults were more likely to undergo an OP approach than non-older adult patients. This supports our hypothesis of potential avoidance of surgical intervention in older adult patients, which typically have more comorbidities and thus a higher CCI, consistent with our data. The fundamental concept of organ preservation has been a part of the surgical praxis for rectal cancer as early as the 1980 s but without meaningful progress until the development and use of contemporary systemic and radiation therapy regimens [12]. These regimens lead to a cCR in a proportion of patients with locally advanced rectal cancer or partial clinical response allowing for local excision and an organ-preserving approach [13, 14]. Given this evidence, patients with more advanced stage II and III tumors were being offered neoadjuvant therapy in hopes of achieving cCR and organ preservation [2]. On the other hand, patients with high risk T1 and T2 lesions were recommended oncologic resection, resulting in an APR or LAR with coloanal anastomoses for distal rectal tumors.

This treatment paradox is beginning to change. The CARTS study examined patients with T1-3N0 lesions. Patients received long course neoadjuvant chemoradiation and were able to achieve organ preservation 74% of the time [14]. More recent early phase II trial results have demonstrated effectiveness of an OP approach for T1-3ab lesions [1]. The most recent version of the National Comprehensive Cancer (NCCN) guidelines for rectal cancer considers neoadjuvant therapy an option for select cases of distal T1/2 lesions [1, 5].

The differences seen in the current study with regard to age and the disparity observed in OPR are likely partially due to the concern for morbidity of oncologic resection in the more advanced age group. This is likely secondary, in part, to an increased number of comorbidities in older adult patients as was seen in the study. The subgroup analysis further demonstrated the impact of comorbidities on OP and the avoidance of major oncologic resection. Although advanced age introduces added risk for any modality of therapy, advances have been made in both the realm of surgery and systemic therapy to minimize complications. Specifically in rectal cancer, retrospective studies have shown that a substantial proportion of older adult patients do successfully complete neoadjuvant therapy [5, 15]. In fact, early evidence suggests that younger patients have more aggressive tumors that do not respond as well to neoadjuvant therapy [16]. While major resection has been shown to have favorable outcomes and equivalent complication rates in older adult patients compared to younger populations, the risks and benefits of organ preservation vs oncologic resection are relevant regardless of age [16, 17].

Racial/ethnic disparities along the continuum of care for colorectal cancer have been shown but data specific to OP have not yet been examined [18]. Although the present study would suggest non-Hispanic Black patients have an increased odds of receiving OP, it is not possible to clarify if this is more illustrative of known racial-ethnic disparities in the receipt of surgical resection rather than true differences in organ preservation rates (OPR).

There is little evidence evaluating the influence of facility type on OPR. A SEER-based study did not find hospital variation as a significant influence on sphincter preservation in older adult patients with stage I–III rectal cancer [19]. The patterns of treatment also differ for locally advanced rectal cancer. A greater preponderance of total neoadjuvant therapy is delivered at academic centers, with a time trend of increasing OPR as well [2, 20]. Interestingly, the odds of receiving OP in the current study were higher in a community cancer hospital than in an academic hospital. This finding is in contrast to other studies, which report increased rates of OP in high volume specialized centers [21, 22]. Further research is needed to investigate this disparity.

This study has known limitations. First, patient data in the NCDB is not population-based and does not broadly represent all facilities and their patients as all hospitals are accredited by the Commission on Cancer limiting the generalizability and inherently underrepresents rural populations. However, when patients with rectal cancer are treated at a specialized cancer center, outcomes are improved, and thus, an argument can be made for the treatment of all patients with rectal cancers at a specialized cancer center [23, 24]. Second, data is entered retrospectively and limited to variables collected by cancer registrars. This likely introduces bias and the opportunity for reporting errors that is inherent to large databases [25]. Third, any confounding factors not tracked as a variable in the database cannot be assessed or controlled for. Fourth, the exact rational for type of therapy cannot be determined. The actual receipt of therapy in this study defines the treatment outcome but cannot differentiate between patients who may have received neoadjuvant care with the intent of TME but ultimately refused surgery or developed progression of disease precluding surgical treatment, therefore, becoming inaccurately classified as receiving OP. However, this likely represents a very small proportion of the cohort [26]. Fifth, the definition used for older adults is arbitrary. Although others have used various age cutoffs, our data can only be extrapolated to those 70 years of age and older. Sixth, current recommendations for an OP approach are reserved for those with distal rectal cancers. Because of limitations of the NCDB, it is not known how many of the patients in each cohort had upper, middle, and distal cancer. Finally, it is worth mentioning that with advancements in systemic therapy, treatment of high-risk stage I rectal cancer is likely to undergo the same changes at stage II/III with a higher percentage of patients being treated with organ preserving approaches as studies are showing its efficacy in this patient population [1]. Upcoming results from the NEO-RT trial specifically studying the high-risk T1 and T2 population will continue to add to the changing landscape [27].

## Conclusion

The treatment of rectal cancer is rapidly changing. Although current recommendations for T1 lesions with high-risk features and T2 lesions are oncologic resection, this results in increased surgical morbidity, including higher rates of low anterior resection syndrome and possibly a permanent stoma. Our analysis indicates that older adult patients were more likely to undergo an OP approach, likely as result of these complications and increased number of comorbidities in this age group. Current and ongoing studies have the potential to change the treatment paradigm in all patients with high-risk stage I rectal cancer.

## Data Availability

The National Cancer Database (NCDB) does not allow sharing of data.

## Declarations

### Meeting

This study was presented as an ePoster at the 125th Annual Scientific Meeting of the American Society of Colon and Rectal Surgeons in Baltimore, Maryland, June 1–4, 2024.
